# AFM signal model for dysarthric speech classification using speech biomarkers

**DOI:** 10.3389/fnhum.2024.1346297

**Published:** 2024-02-20

**Authors:** Shaik Mulla Shabber, Eratt Parameswaran Sumesh

**Affiliations:** School of Electronics Engineering, VIT-AP University, Amaravati, Andhra Pradesh, India

**Keywords:** dysarthric speech, ALS, machine learning, speech signal classification, amplitude and frequency modulation, bulbar motor dysfunction

## Abstract

Neurological disorders include various conditions affecting the brain, spinal cord, and nervous system which results in reduced performance in different organs and muscles throughout the human body. Dysarthia is a neurological disorder that significantly impairs an individual's ability to effectively communicate through speech. Individuals with dysarthria are characterized by muscle weakness that results in slow, slurred, and less intelligible speech production. An efficient identification of speech disorders at the beginning stages helps doctors suggest proper medications. The classification of dysarthric speech assumes a pivotal role as a diagnostic tool, enabling accurate differentiation between healthy speech patterns and those affected by dysarthria. Achieving a clear distinction between dysarthric speech and the speech of healthy individuals is made possible through the application of advanced machine learning techniques. In this work, we conducted feature extraction by utilizing the Amplitude and frequency modulated (AFM) signal model, resulting in the generation of a comprehensive array of unique features. A method involving Fourier-Bessel series expansion is employed to separate various components within a complex speech signal into distinct elements. Subsequently, the Discrete Energy Separation Algorithm is utilized to extract essential parameters, namely the Amplitude envelope and Instantaneous frequency, from each component within the speech signal. To ensure the robustness and applicability of our findings, we harnessed data from various sources, including TORGO, UA Speech, and Parkinson datasets. Furthermore, the classifier's performance was evaluated based on multiple measures such as the area under the curve, F1-Score, sensitivity, and accuracy, encompassing KNN, SVM, LDA, NB, and Boosted Tree. Our analyses resulted in classification accuracies ranging from 85 to 97.8% and the F1-score ranging between 0.90 and 0.97.

## 1 Introduction

Dysarthria, a prominent and intricate motor speech disorder, originates from malfunctions within speech production subsystems or coordination issues due to neurological damage. This neuro-motor condition results from neurological damage that intricately affects the motor components of speech production. These manifestations include diminished vocal volume, imprecise articulation, disturbances in coordinating respiratory and phonatory subsystems, and the presence of irregular speech pauses. The amalgamation of these defining attributes underscores the multifaceted nature of this speech disorder (Joshy and Rajan, 2022).

Dysarthria represents a complex range of speech impairments, and its underlying causes can vary widely. Understanding and addressing specific characteristics and the etiology of dysarthria are crucial in developing effective intervention strategies, thereby improving the quality of life for individuals affected by this disorder. Due to a variety of distinguishing characteristics, including decreased vocal tract volume, restricted tongue flexibility, changed speech prosody, imprecise articulation, and erratic fluctuations in speech rate, dysarthric speech is typically difficult to understand (Narendra and Alku, 2019). These factors collectively contribute to comprehension challenges in dysarthric communication. Evaluating dysarthric speech becomes imperative to distinguish it from typical speech. This evaluation serves as a crucial diagnostic step to differentiate healthy speech patterns from those indicative of dysarthria. Typically, speech evaluation is conducted through conventional methods, often by speech-language pathologists. These professionals administer intelligibility tests to assess the presence and severity of dysarthria. Through these evaluations, healthcare providers gain comprehensive insights into the nature and extent of the speech disorder, enabling tailored interventions and treatment strategies for individuals affected by dysarthria (Ramanarayanan et al., 2022).

This condition is most commonly associated with neurological injuries or diseases, such as CP, brain tumors, strokes, and brain injuries. Additionally, it can manifest as a symptom of various neurodegenerative diseases like PD and ALS. One of the major characteristics of dysarthria is its significant impairment on speech clarity. This reduced speech intelligibility primarily stems from a group of speech-related deficits, including decreased speech pace, irregular speech prosody, restricted tongue flexibility, poor articulation, and reduced vocal tract volume. These features collectively pose considerable challenges for individuals with dysarthria and those trying to comprehend their speech (Duffy, 2012; Narendra and Alku, 2019). In the field of speech signal processing, there is a growing acknowledgment of the significance of using speech-based biomarkers as a means to gain insights into neurological health conditions. Modern investigations have explored the potential of speech analysis as a biomarker to detect a range of neurological disorders and mental health conditions. This development holds great promise for enhancing disease identification and diagnostic procedures (Ramanarayanan et al., 2022).

The analysis of speech patterns in individuals with cerebral palsy has yielded promising outcomes for the early identification and continuous monitoring of neurological conditions like ALS and PD (Hecker et al., 2022). These encouraging results can be attributed to noticeable alterations in speech and voice characteristics, including a decrease in speech rate and an increase in vocal intensity. The onset of slurred speech often serves as one of the initial indications of these conditions. Leveraging speech analysis to detect these variations may offer the potential to identify individuals with these disorders in their early stages and to track the progression of the diseases over time (Koops et al., 2023).

Speech, as a signal, exhibits non-stationary characteristics. A non-stationary speech signal is characterized by fluctuating amplitude and frequency components, making a modulated signal more suitable for the analysis of such non-stationary signals. To effectively observe distinct variations in amplitude and changes in frequency, the utilization of independent modulated frequency and amplitude signal model is considered as a potential tool. The AFM signal model integrates both amplitude modulation (AM) and frequency modulation (FM) signal models, presenting an improved option for effectively portraying amplitude and frequency fluctuations in non-stationary speech signals (Bansal and Sircar, 2019). The segregation of distinct components within multi-component speech is a pivotal stage in speech signal analysis, encompassing various attributes such as frequency, phase, and amplitude. In this paper, the approach employed for feature extraction from recorded speech phonemes utilizes the AFM signal decomposition model. This particular model has previously undergone application and testing across diverse speech-processing contexts. The multi-component multitone AFM signal model proves to be well-suited for feature extraction in the analysis of both voiced and unvoiced speech phonemes (Bansal and Sircar, 2018).

This paper is structured as follows: Section 2 covers the Literature survey, and Section 3 outlines the discusses the methodology, Classification techniques and Feature extraction, while Section 4 explores classification techniques and performance measures, and presents results. Section 5 provides comparisons with other approaches, while Section 6 presents the conclusions of the paper. In addition, we have included a comprehensive list of abbreviations in Table 1 for reference.

**Table 1:** List of abbreviations.

**S.No**.	**Abbreviation**	**Definition**
1	FB	Fourier-Bessel
2	NB	Naive Bayes
3	ALS	Amyotrophic lateral sclerosis
4	LDA	Linear Discriminant Analysis
5	CP	Cerebral palsy
6	PD	Parkinson's disease
7	KNN	K-Nearest Neighbors
8	AM	Amplitude modulation
9	FM	Frequency modulation
10	AFM	Amplitude and frequency modulated
11	DESA	Discrete Energy Separation Algorithm
12	AE	Amplitude Envelope
13	IF	Instantaneous Frequency
14	DYS	Dysarthric Speech
15	HC	Healthy control
16	MFCCs	Mel-frequency cepstral coefficients
17	TP	True positive
18	TN	True negative
19	FN	False negative
20	FP	False positive
21	SVM	Support Vector Machine
22	ANN	Artificial Neural Network
23	DDK	Diadochokinetic rate

## 2 Literature survey

One of the key challenges in assessing the severity of different dysarthria types is the absence of comprehensive analyses derived from a diverse pool of speakers encompassing various dysarthria types and differing degrees of severity. Furthermore, the presence of numerous distinct dysarthria varieties adds to the complexity of this issue (Kim et al., 2011). In the pursuit of identifying dysarthric speech, the author exploited the power of neural networks. These networks were applied to centroid formants, which represent extended speech characteristics, aiding in the discrimination between dysarthric and non-dysarthric speech. Subsequently, the study employed an experimental database consisting of 200 speech samples from ten individuals with dysarthria and an equal number of speeches from ten age-matched healthy individuals (Ijitona et al., 2017). Mani et al. created a software program capable of making determinations about specific features through the application of fractal analysis. This was achieved by utilizing both acoustic and connected articulatory sound recordings from the speech under examination. The classification method of choice in their study was the Diadocho kinetic test (Spangler et al., 2017).

Moro-Velazquez et al. conducted an extensive investigation into the assessment of PD through computerized analysis of speech signals, focusing on phonatory and articulatory factors. Their review encompassed a broad spectrum of areas, and their findings led to the conclusion that the severity of PD is indeed correlated with challenges in both phonation and articulation (Moro-Velazquez et al., 2021). Vásquez-Correa et al. (2019) employed MFCCs for the classification of individuals with PD and HC. They conducted this classification using the Spanish PC-GITA database. The authors employed SVMs and utilized statistical functionals derived from MFCCs. These coefficients were computed on the Bark bands and were extracted from various speech sources, including individual utterances, DDK tasks, text reading, and monolog segments. The MFCCs were computed specifically on the Bark frequency scale.

Phonation, a common speech task employed to appraise the condition of the phonatory speech subsystem, enables the evaluation of diverse facets of an individual's voice. In this work, the authors investigated the application of cepstral analysis to distinguish individuals with PD from those with alternative neurological disorders. The researchers gathered vocal recordings from 50 participants and subsequently examined these recordings through the application of three distinct cepstral methodologies. The most favorable outcome reached entailed a remarkable 90% accuracy, accomplished by employing the first 11 coefficients of the PLP, coupled with linear SVM kernels. This investigation carries significant implications for advancing the diagnosis of PD and other neurological conditions (Benba et al., 2016).

Vashkevich and Rushkevich (2021) introduced a methodology for voice assessment within automated systems, aimed at distinguishing individuals without ALS from those afflicted with the condition. The main focus of the research is on using acoustic analysis of sustained vowel phonations to classify patients with ALS. Through the use of MFCC parameters, the authors have determined that the spectral envelopes of the vowels /a/ and /i/ include essential markers for the early identification of ALS.

## 3 Methodology

**Figure 1:**
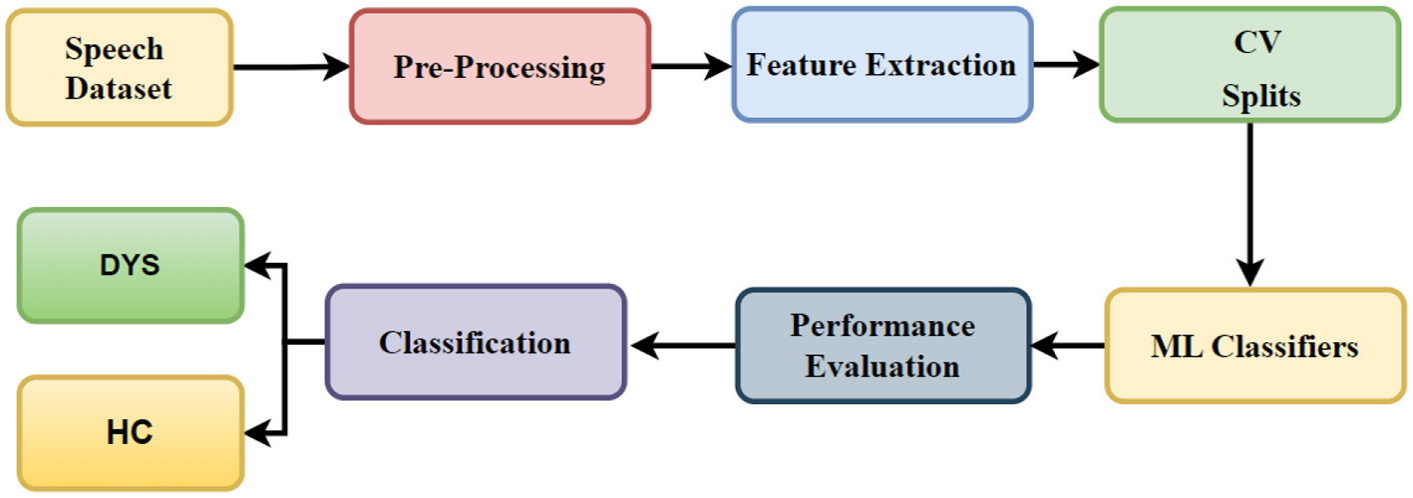
Workflow of the proposed system.

In Figure 1, the workflow of the proposed system is presented. The proposed methodology for the detection of Dysarthic speech (DYS) comprises a series of discrete phases, including the establishment of a phonetic database derived from speech, the initial processing of acquired data, the extraction of salient characteristics, and the subsequent differentiation of individuals with Dysarthic speech from those constituting the healthy control (HC) group. In the primary stage, we systematically compile the requisite phonetic elements from speech samples procured from both Dysarthic speech-affected individuals and their HC counterparts.

### 3.1 Database

To ensure clarity and minimize any potential ambiguities in the establishment of our phoneme database, we have carefully verified and merged many pre-existing databases. We undertook this compilation process to minimize any doubts about the content and structure of the dataset and to provide a solid basis for our work. To assess the validity of the proposed study, we used data from established dysarthric databases, namely, the TORGO database (Rudzicz et al., 2012), the Universal Access dysarthric speech corpus (UA-Speech) (Kim et al., 2008), and the PD database (Viswanathan et al., 2019). The TORGO database encompasses utterances from seven individuals without speech disorders and eight patients with dysarthria. The UA-Speech database is inclusive of speech samples contributed by both unaffected individuals (totaling 13) and those afflicted by dysarthria (totaling 19). Additionally, 22 healthy controls and twenty-four patients with Parkinson's disease (PD) diagnosed in the ten years prior were gathered from Monash Medical Center's Movement Disorders Clinic to assemble the PD database.

We collected data from several datasets, a total of 4,900 vowel phonemes for our study. Within this phoneme dataset, 2,450 were sourced from individuals exhibiting healthy speech phonemes, while an equivalent count of 2,450 originated from individuals affected by dysarthria. Subsequently, we focused on two specific datasets derived from this pool. The first dataset exhibits a balanced distribution, comprising an equal number of samples from both healthy and dysarthric speech sources. Conversely, the second dataset represents an imbalanced distribution, where the ratio between dysarthric and healthy samples stands at 7:3, consisting of 2,450 instances of dysarthric phonemes and 1,050 instances of healthy phonemes. This latter dataset, denoted as Dataset 2, forms the basis for our analysis. The description of this clearly presented in the below Description of Balanced and Imbalanced Datasets Table 2.

**Table 2:** Description of balanced and imbalanced datasets.

**Dataset**	**Dysarthric speech phonemes**	**Healthy speech phonemes**	**Total phoneme samples**
Balanced (Dataset 1)	2,450	2,450	4,900
Imbalanced (Dataset 2)	2,450	1,050	3,500

#### 3.1.1 Pre-processing

In TORGO dataset, words have been identified with the starting and ending positions of phonemes. We segmented these words to extract individual phonemes, subsequently utilized these segmented phonemes for our analysis. Conversely, for the other datasets (UA-Speech and PD database) we conducted phoneme segmentation using Praat (Boersma and Weenink, 2001), a robust speech analysis software, which served as an important pre-processing step. Additionally, certain pre-segmented phonemes were included in these datasets, and no pre-processing steps were applied in the proposed system.

### 3.2 Feature extraction

After the pre-processing stage, feature extraction and the classification of individuals into DYS or HC groups are performed. For feature extraction, we employed an AFM signal model to analyze speech phonemes. This involved extracting features like the amplitude envelope (AE) and instantaneous frequency (IF) functions using the Fourier-Bessel (FB) expansion and the discrete energy separation algorithm (DESA). The FB expansion helped separate the individual components of the speech phoneme, with two components considered in our study. In speech, it's common practice to focus on the first two or three components of a speech phoneme because they usually hold the most important information about the phoneme's characteristics (Bansal and Sircar, 2019). Therefore, in our study, we focused on analyzing two components of the speech phoneme. The DESA algorithm was then applied to each component to extract the AE and IF functions. From the AE function, parameters such as amplitude, modulation index (μ_*a*_), modulating angular frequency (ω_*a*_), and modulating phase (θ_*a*_) of the amplitude modulation segment are obtained. Similarly, the IF function yields parameters, including carrier frequency (ω_*c*_), modulation index (μ_*f*_), modulating angular frequency (ω_*f*_), and modulating phase (θ_*f*_) of the frequency modulation segment (Bansal and Sircar, 2021, 2022). These parametric representation employed aligns with an AFM signal model (Pachori and Sircar, 2010; Bansal and Sircar, 2018, 2019), as speech signals do not display stationary characteristics (Sircar and Syali, 1996; Sircar and Sharma, 1997; Upadhyay et al., 2020). These features were utilized for the analysis and synthesis of speech phonemes. In total, we extracted 28 features from the two components, with each component contributing 14 distinct features. This approach enabled us to obtain 28 features from the two components of each phoneme. Each component, namely Component 1 and Component 2, contributed 14 distinct features, resulting in a combined set of 28 features utilized for our analysis. The details of these features extracted from the two components are presented in Table 3.

**Table 3:** Features of component.

Amplitude envelope	*A*	μ_*a*1_	μ_*a*2_	ω_*a*1_	ω_*a*2_	θ_*a*1_	θ_*a*2_
Instantaneous frequency	ω_*c*_	μ_*f*1_	μ_*f*2_	ω_*f*1_	ω_*f*2_	θ_*f*1_	θ_*f*2_

**Figure 2:**
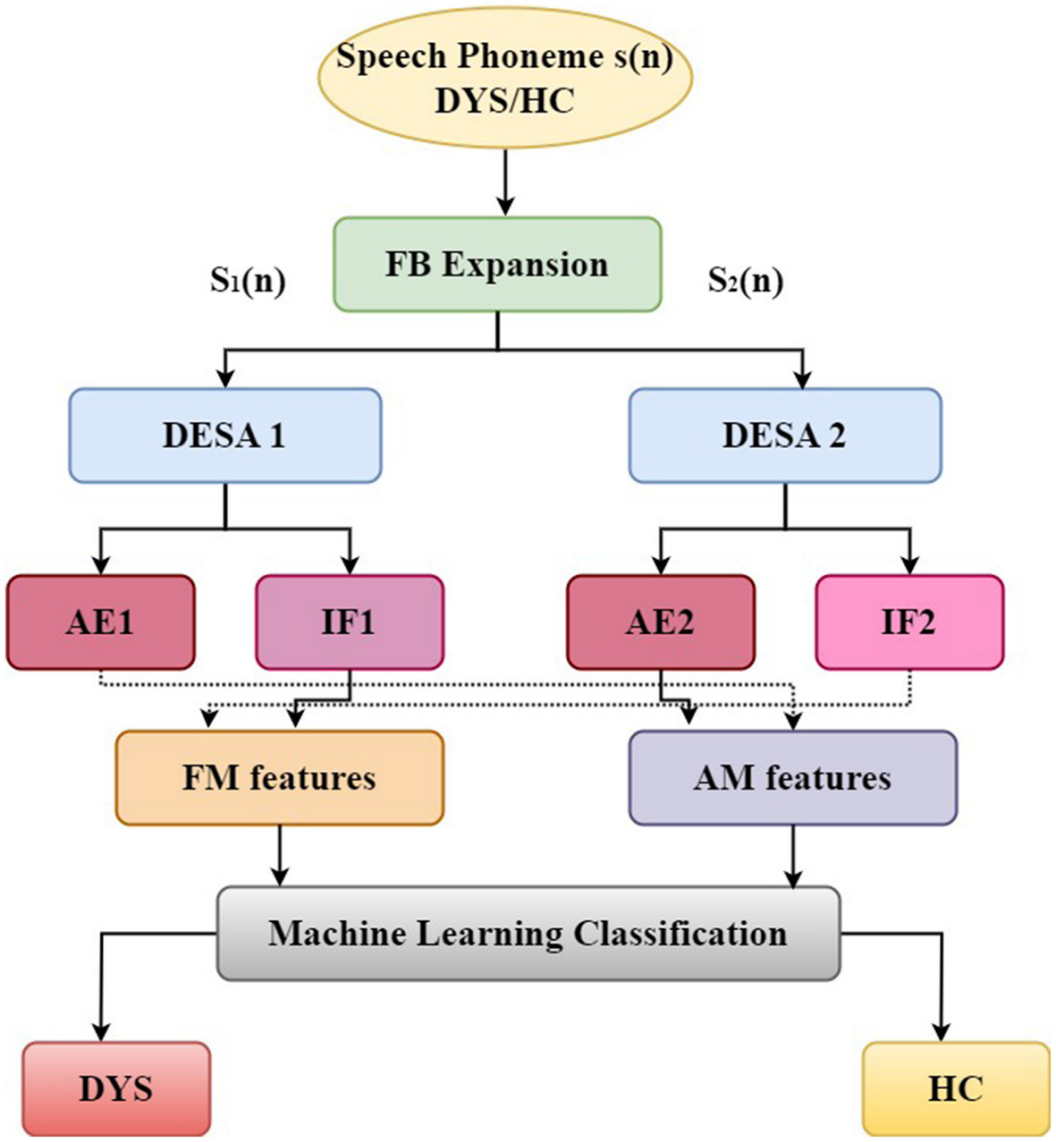
A schematic diagram of the proposed DYS/HC classification.

Figure 2 provides a detailed flow diagram of feature extraction and classification. The subsequent sections will delve into a detailed breakdown of the suggested strategy. Initially, an AFM signal model is introduced for subsequent signal analyses. Following this, the subsequent subsections detail the technique for extracting modulating features utilized in the proposed methodology, culminating in a binary classification approach for detecting DYS/HC. This binary classification is aimed at determining the presence of DYS or HC.

### 3.3 AFM signal model

Non-stationary signals, represented by speech signals (Sircar and Syali, 1996; Sircar and Sharma, 1997), can be effectively represented as a sum of sinusoidal functions through techniques like Fourier analysis. This decomposition process disaggregates non-stationary signals into their constituent frequency components. These frequency components can be modeled using sinusoidal functions with varying frequencies and amplitudes, forming a multi-tone AFM signal model (Bansal and Sircar, 2019). The parametric representation of a non-stationary signal *s[n]* is presented in Equation (1), which utilizes both two-tone AM and two-tone FM signals (Equation 2).


(1) $$\begin{array}{l} s\left[n\right]=s_{1}\left[n\right]+s_{2}\left[n\right] \end{array}$$

(2) $$\begin{array}{l} \begin{array}{cc} s_{i}\left[n\right]= & \;A\left(1+\mu_{a1}^{i}\cos\left(\omega_{a1}^{i}n+\theta_{a1}^{i}\right)+\mu_{a2}^{i}\cos\left(\omega_{a2}^{i}n+\theta_{a2}^{i}\right)\right)\times \\ \;\cos\left(\omega_{c}^{i}n+\mu_{f1}^{i}\sin\left(\omega_{f1}^{i}n+\theta_{f1}^{i}\right)+\mu_{f2}^{i}\sin\left(\omega_{f2}^{i}n+\theta_{f2}^{i}\right)\right) \end{array} \end{array}$$

where *i*=1,2; *A* is the amplitude; ω_*c*_ is the carrier frequency of the signal model; ω_*a*1_, ω_*a*2_, ω_*f*1_, ω_*f*2_ are the modulating angular frequencies;, μ_*a*1_, μ_*a*2_, μ_*f*1_, μ_*f*2_ are the modulation indexes; θ_*a*1_, θ_*a*2_, θ_*f*1_, θ_*f*2_ are the modulating phase of the modulated signal.

#### 3.3.1 Mono component separation of speech signal

Fourier-Bessel expansion is a mathematical method that effectively decomposes a signal into its constituent frequency components. Speech, a complex signal composed of various frequency components, can be effectively analyzed and studied using this technique. By employing FB expansion, individual frequency components of speech can be isolated and examined in detail. This expansion is a mathematical technique that allows the representation of a signal as a sum of components, each characterized by its own amplitude and frequency. In the context of speech signal processing, the Fourier-Bessel expansion is applied to decompose the multicomponent speech signal into its constituent components, thereby isolating the individual elements that contribute to the overall phoneme (Bansal and Sircar, 2019; Upadhyay et al., 2020). The expansion of the FB series is provided (Equations 3 and 4).


(3) $$\begin{array}{l} S\left[t\right]=\overset{P}{\underset{p=1}{\sum }}C_{p}J_{0}\left(\frac{\lambda_{p}t}{T}\right) \end{array}$$

The FB coefficients *Cp* can be calculated as


(4) $$\begin{array}{l} C_{p}=\frac{2*I}{T^{2}{\left[J_{1}\left(\lambda_{p}\right)\right]}^{2}} \end{array}$$

$$\begin{array}{l} \text{where}\;I=\overset{T}{\underset{0}{\int }}t\;S\left(t\right)\;J_{0}\left(\frac{\lambda_{p}t}{T}\right)dt \end{array}$$

where *T* is the period of signal, λ_1_, λ_2_, λ_3_...........λ_*p*_; *p* = 1, 2, ..., *P* are the complex positive roots of *J*_0_(λ) = 0 in the increasing order and *J*_*l*_(.) is the *l*^*th*^-order Bessel function for *l* = 0 and 1.

#### 3.3.2 Discrete energy separation algorithm

DESA, as a methodology, serves the purpose of dissecting a non-stationary speech signal into its constituent components, namely the AE and the IF. This separation is achieved by distinguishing and subsequently analyzing distinct amplitude and frequency bands. This methodology involves the estimation of the AE and IF (Upadhyay et al., 2017) function parameters by employing DESA on a segmented phonemic component of the speech signal. In the proposed method, we consider a dual-tone amplitude and frequency modulation. By replacing the sinusoidal variation with its corresponding complex exponentials, it becomes possible to extend the estimation of feature vectors for the AE and IF. Concerning the amplitude parameters, (Equation 5) illustrates the representation of the amplitude envelope extracted by the DESA (Pachori and Sircar, 2010; Bansal and Sircar, 2018, 2019) within the signal model (Equation 1).


(5) $$\begin{array}{l} a\left[n\right]=A\left(1+\frac{1}{2}\left[E_{1}+E_{2}\right]\right)\\ \text{where}\;E_{1}=\mu_{a1}\left[e^{j\theta_{a1}}\;e^{j\omega_{a1}n\;}+e^{-j\theta_{a1}}\;e^{-j\omega_{a1}n\;}\right]\\ \text{and}\;E_{2}=\mu_{a2}\left[e^{j\theta_{a2}}\;e^{j\omega_{a2}n\;}+e^{-j\theta_{a2}}\;e^{-j\omega_{a2}n\;}\right]\;\text{for}\;n=0:N-1 \end{array}$$

and (Equation 6) illustrates the representation of the instantaneous frequency extracted by the DESA of the signal model (1) is


(6) $$\begin{array}{l} \omega \left[n\right]=\omega_{c}n+\frac{1}{2}\left[F_{1}+F_{2}\right]\\ \text{where}\;F_{1}=\mu_{f1}\omega_{f1}\left[e^{j\theta_{f1}}\;e^{j\omega_{f1}n\;}+e^{-j\theta_{f1}}\;e^{-j\omega_{f1}n\;}\right]\;\text{and}\\ F_{2}=\mu_{f2}\omega_{f2}\left[e^{j\theta_{f2}}\;e^{j\omega_{f2}n\;}+e^{-j\theta_{f2}}\;e^{-j\omega_{f2}n\;}\right]\text{for}\;n=0:N-1 \end{array}$$

**Figure 3:**
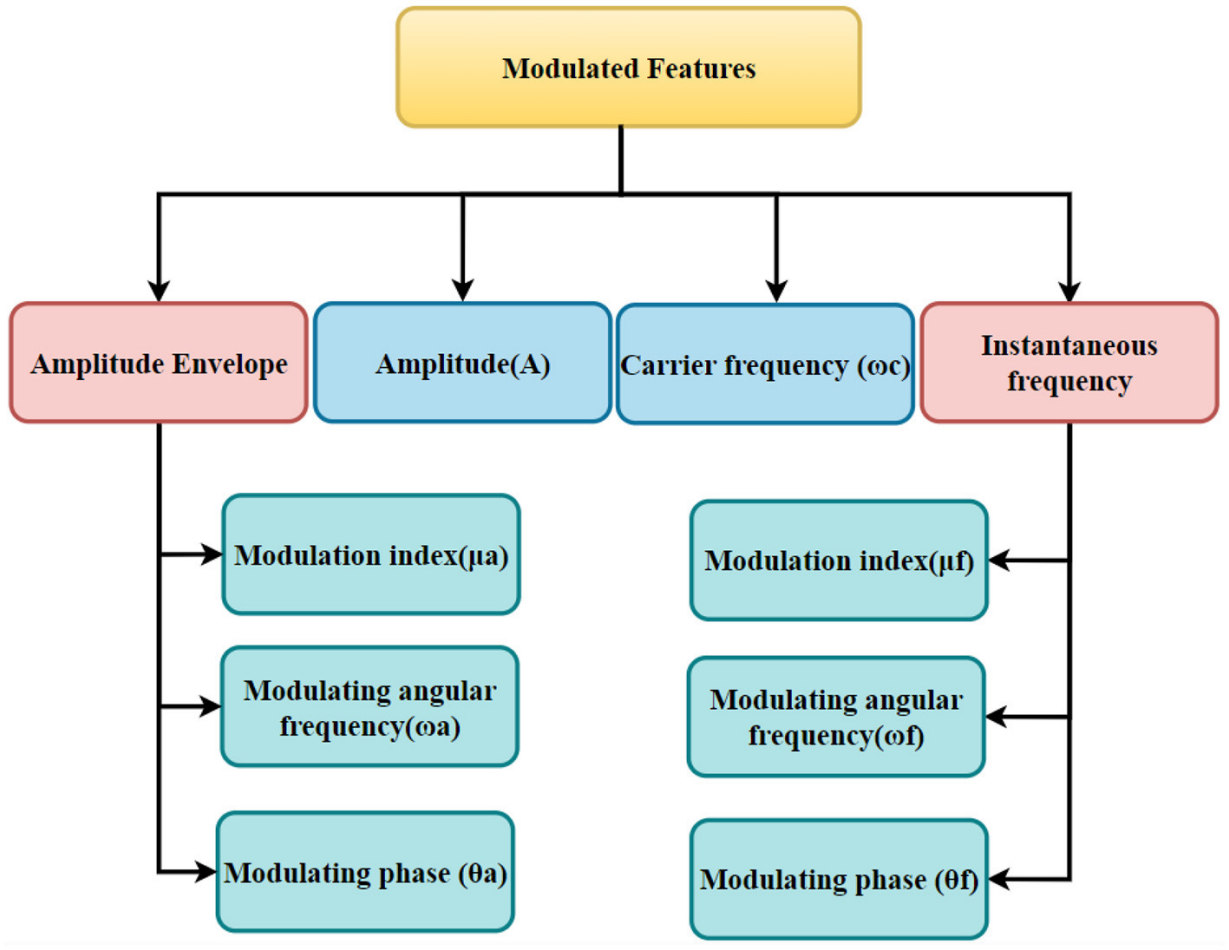
A structured illustration of the extracted modulated features.

We employed FB-DESA to estimate parameters for recovering the amplitude envelope and instantaneous frequency functions from the signal components. The parameters obtained from the amplitude envelope encompass amplitude (*A*), modulation index (μ_*a*_), modulating angular frequency (ω_*a*_), and modulating phase (θ_*a*_) of the amplitude modulation section. Correspondingly, parameters retrieved from the instantaneous frequency involve carrier frequency (ω_*c*_), modulation index (μ_*f*_), modulating angular frequency (ω_*f*_), and modulating phase (θ_*f*_) of the frequency modulation part. We take into account the following features for the feature extraction: The amplitude, carrier frequency, and modulation frequencies (AM and FM) of the tone of the AE and IF spectra of each phoneme are the features employed in this research. A structured illustration of the extracted modulated features is presented in Figure 3.

### 3.4 Machine learning

Machine learning classifiers play a pivotal role in data analysis, facilitating well-informed decision-making and predictive tasks. This research inquiry delves into the attributes and practical applications of some prominent classifiers: LDA, NB, SVM, KNN, and Boosted tree. These classifiers exhibit unique characteristics and underlying assumptions, which render them well-suited for diverse types of data and problem contexts. By unraveling the intricacies of these classifiers, this study seeks to enhance our understanding of their capabilities and their respective domains of applicability within the field of data analysis, contributing to a comprehensive perspective on the utilization of machine learning classifiers in real-world scenarios.

In our study, we utilized two distinct datasets: Dataset 1 comprised 2,450 samples each of dysarthric and healthy speech, while Dataset 2 consisted of 2,450 samples of dysarthric speech and 1,050 samples of healthy speech as discussed in Section 3.1.

The selection of SVM, Naive Bayes, KNN, LDA, and ensemble boosted tree classifiers was motivated by the unique characteristics of these datasets. Specifically, the larger size and balanced nature of Dataset 1 allowed for a comprehensive evaluation of classifier performance under equal class distribution. On the other hand, the imbalanced nature of Dataset 2 provided an opportunity to assess the classifiers' robustness in handling uneven class proportions, simulating a more real-world scenario. The suitability of individual classifiers for the datasets used is summarized below:

- SVM's capacity to handle high-dimensional data (Sun et al., 2022) and effective class separation made it a fitting choice for Dataset 1's balanced classes.
- NB and KNN were selected for their simplicity and non-parametric nature, accommodating Dataset 2's imbalanced structure and varying feature complexities (Venkata Subbarao et al., 2021; Ramesh et al., 2023).
- LDA's role in dimensionality reduction (Haulcy and Glass, 2021) and preserving class discriminatory information was crucial for both datasets, contributing to effective feature representation.
- The ensemble-boosted tree classifiers were included to address the relationships within the datasets and to mitigate potential overfitting concerns (Sisodia et al., 2020).

## 4 Results and discussion

In this research, machine learning classifiers were utilized to analyze features extracted from a AFM signal model. These classifiers aimed to identify and categorize the various forms of dysarthric speech observed in each participant (dysarthric speech exhibited by individuals with ALS, PD, and CP). ALS patients typically experience slurred speech and difficulty controlling the pitch of their voice, leading to noticeable variations in amplitude. Individuals with PD often exhibit reduced loudness, monotone speech, and hesitations in their speech patterns. CP patients often demonstrate speech characterized by imprecise articulation, variations in speech rate, and inconsistent speech rhythm. These distinct speech characteristics in ALS, PD, and CP individuals serve as differentiating factors between these patient populations and healthy individuals.

We used three different approaches to enable a robust evaluation of the model's generalizability and to avoid potential biases caused by specific cross-validation techniques:

- Split ratio: to begin, we divided the dataset into training and testing sets using 80:20 and 70:30 splits, respectively, to provide preliminary insights into model performance.
- K-fold cross-validation: we then used five-fold and 10-fold cross-validation, a popular method that randomly divides the data into five and 10 folds, each of which serves as a test set once.
- Leave-one-subject-out cross-validation (LOSO CV): because of the possibility for dependencies within data belonging to the same subject, we additionally developed LOSO CV, which iteratively trains the model on all data except that belonging to a single subject, ensuring subject-independent evaluation. In our study, we considered a balanced dataset (84 subjects) comprising 42 healthy controls and 42 dysarthic subjects. Furthermore, for the imbalanced dataset (63 subjects) we have taken into account 42 dysarthic subjects and 21 healthy subjects.

Further, we present the performance evaluation of various classifiers in the context of binary classification. These classifiers have been assessed using a set of key metrics, shedding light on their effectiveness in distinguishing between positive and negative instances. The following metrics can be used to determine how well the database performs when using the AFM signal model mentioned in the above section.

In our work, we have chosen to employ the accuracy (Acc) metric as one of the key methods for assessing the classification model's effectiveness. However, it's important to highlight that solely relying on accuracy might present a somewhat limited view of the classifier's overall performance. To gain a better understanding, we also considered other metrics like precision, recall, F1-score, and area under the receiver operating characteristic curve (AUC). The performance metrics regarding the proposed work are depicted in Tables 4–**8** provided below.

**Table 4:** Balanced dataset split results.

**SNo**.	**Classifier**	**Split**	**Train (%)**	**Test (%)**	**Precision**	**Recall**	**F1 score**	**AUC**
1	LDA	80:20	84.11	84.18	0.8623	0.8025	0.8313	0.8407
		70:30	84.02	84.69	0.8651	0.8104	0.8368	0.8458
2	NB	80:20	77.93	76.63	0.8049	0.6849	0.7401	0.7641
		70:30	78.05	77.62	0.8165	0.6938	0.7502	0.7737
3	KNN	80:20	88.34	85.92	0.8912	0.8088	0.8480	0.8578
		70:30	88.10	84.56	0.8714	0.7992	0.8337	0.8442
4	SVM	80:20	89.21	83.16	0.8711	0.7668	0.8156	0.8298
		70:30	89.71	82.24	0.8574	0.7598	0.8057	0.8205
5	Boosted tree	80:20	99.92	93.78	0.9387	0.9328	0.9357	0.9376
	70:30	99.85	91.56	0.9095	0.9171	0.9133	0.9157

This investigation conducted an in-depth evaluation of a signal model based feature extraction technique applied to speech signals, yielding 28 distinct features. The analysis encompassed two primary scenarios: balanced and imbalanced datasets. Each scenario was further subjected to rigorous testing under varying train-test split ratios (80:20 and 70:30) and cross-validation configurations (five-fold, 10-fold and LOSOCV).

Performance under balanced dataset conditions presented in Table 4 revealed consistent and commendable metrics across classifiers, irrespective of split ratios. Performance measures such as accuracy, precision, recall, F1 scores, and AUC values remained stable. This finding underscores the technique's proficiency in distinguishing speech patterns when data distribution is balanced.

Introducing data imbalance presented in Table 5 (2,450 Dysarthic speech/1,050 healthy speech), led to discernible variations in performance metrics, particularly precision, recall, and AUC scores. Certain classifiers exhibited sensitivity toward the minority class (healthy speech), demonstrating a tendency to favor the dominant class. Others encountered challenges in maintaining robust performance due to data skewness.

**Table 5:** Imbalanced dataset split results.

**SNo**.	**Classifier**	**Split**	**Train (%)**	**Test (%)**	**Precision**	**Recall**	**F1 score**	**AUC**
1	LDA	80:20	93.00	94.43	0.9435	0.8520	0.8954	0.9161
		70:30	93.63	92.48	0.9291	0.8058	0.8631	0.8901
2	NB	80:20	89.57	90.71	0.7937	0.9031	0.8449	0.9059
		70:30	90.00	89.71	0.8150	0.8414	0.8280	0.8809
3	KNN	80:20	95.21	95.00	0.8966	0.9286	0.9123	0.9435
		70:30	95.76	93.24	0.8993	0.8673	0.8830	0.9134
4	SVM	80:20	89.21	83.16	0.8711	0.7668	0.8156	0.8298
		70:30	89.71	82.24	0.8574	0.7598	0.8057	0.8205
5	Boosted tree	80:20	99.6	97.86	0.9840	0.9388	0.9608	0.9664
	70:30	99.1	95.62	0.9519	0.8964	0.9233	0.9388

Balanced-data cross-validation Table 6 revealed consistent and reliable performance metrics across folds for most classifiers, validating the model's resilience and stability. Conversely, in imbalanced data presented in Table 7 situations, while some classifiers maintained high accuracy, precision, recall, and AUC scores, others struggled with data skewness, resulting in variations in performance across folds. This suggests the importance of considering cross-validation to assess how model performance generalizes to unseen data, especially in imbalanced settings.

**Table 6:** Balanced dataset CV results.

**SNo**	**Classifier**	**Fold**	**Train (%)**	**Test (%)**	**Precision**	**Recall**	**F1 score**	**AUC**
1	LDA	Fold 5	88.2	86.33	0.8286	0.8980	0.8904	0.8633
		Fold 10	91	90.57	0.7524	0.9714	0.8272	0.8619
2	NB	Fold 5	77.9	77.14	0.6597	0.8770	0.8351	0.7683
		Fold 10	90.8	90.86	0.8611	0.9298	0.8532	0.8954
3	KNN	Fold 5	88.8	85.51	0.8608	0.8533	0.8540	0.8533
		Fold 10	88.5	86.94	0.8688	0.8717	0.8690	0.8717
4	SVM	Fold 5	87.2	83.27	0.7437	0.9167	0.8939	0.8302
		Fold 10	90.12	87.55	0.8352	0.9238	0.8797	0.8795
5	Boosted tree	Fold 5	95.8	93.06	0.9224	0.9388	0.9378	0.9306
	Fold 10	95.2	94.29	0.9184	0.9673	0.9414	0.9429

**Table 7:** Imbalanced dataset CV results.

**SNo**	**Classifier**	**Fold**	**Train (%)**	**Test (%)**	**Precision**	**Recall**	**F1 score**	**AUC**
1	LDA	Fold 5	93.16	92.86	0.8095	0.9796	0.9444	0.8946
		Fold 10	93.3	90.57	0.7524	0.9714	0.8272	0.8619
2	NB	Fold 5	89.83	89.71	0.8431	0.9194	0.8113	0.8812
		Fold 10	89.97	90.86	0.8611	0.9298	0.8532	0.8954
3	KNN	Fold 5	95.4	95.14	0.9473	0.9340	0.9403	0.9340
		Fold 10	95.2	96.57	0.9621	0.9573	0.9596	0.9573
4	SVM	Fold 5	97.2	92.57	0.7843	0.9839	0.9524	0.8841
		Fold 10	96.3	92.86	0.8148	0.9793	0.8756	0.8971
5	Boosted tree	Fold 5	97.45	96.29	0.996	0.8762	0.9340	0.9381
	Fold 10	98.63	97.82	0.9902	0.9352	0.9640	0.991

These outcomes underscore the pivotal influence of dataset composition on classifier performance. The signal model-based feature extraction technique exhibited robustness and reliability in handling balanced datasets, while its efficacy in addressing imbalanced data presented variable outcomes across different classifiers. These insights yield significant implications for the development and implementation of speech signal classification models, emphasizing the necessity of addressing dataset balance for optimized real-world applications.

The performance metrics of various classifiers, including LDA, NB, KNN, SVM, and Boosted, are displayed in the Table 8. These classifiers were tested using Leave-One-Subject-Out Cross-Validation (LOSO-CV) on two different datasets: one that was balanced (42 subjects of dysarthic and 42 subjects of healthy control speech) and the other that was imbalanced (42 subjects dysarthic speech and 21 subjects healthy control speech). With an accuracy of 91.05%, recall of 0.926, precision of 0.988, and F1-score of 0.949 on the balanced dataset, the Boosted tree stands out for its better performance across all measures. Other classifiers also show strong performance. In contrast, Boosted continues to perform exceptionally well on the imbalanced dataset, demonstrating its resilience to class imbalance with an accuracy of 91.65%, recall of 0.9267, precision of 0.989, and F1-score of 0.946.

**Table 8:** LOSO-CV performance metrics.

**Dataset**	**Classifier**	**Metrics**			
		**Train (%)**	**Test (%)**	**Precision**	**Recall**	**F1-score**
Balanced data	LDA	85.30	84.44	0.946	0.895	0.906
	NB	85.5	85.2	0.996	0.852	0.9164
	KNN	92.23	89.86	0.976	0.898	0.943
	SVM	86.14	82.32	0.974	0.823	0.897
	Boosted	92.80	91.05	0.9880	0.926	0.949
Imbalanced data	LDA	86.51	85.17	0.987	0.851	0.907
	NB	86.5	85.72	0.981	0.867	0.919
	KNN	92.6	87.79	0.916	0.877	0.929
	SVM	87.7	84.36	0.923	0.843	0.903
	Boosted	93.43	91.65	0.989	0.9267	0.946

### 4.1 Accuracy

Accuracy *(Acc)* pertains to a metric that assesses the model's capacity to make accurate predictions relative to the total number of predictions generated (Equation 7). This metric is commonly employed when evaluating classification models to gauge their effectiveness. Accuracy is calculated by considering the proportion of correctly classified samples in relation to the overall sample count, providing insights into the classification system's efficacy. To gain a holistic understanding of the classifier's capabilities, it is advisable to complement the evaluation of accuracy with other performance measures, such as precision, recall, and the F1-score. This multi-dimensional approach ensures a comprehensive appraisal of the classifier's performance.


(7) $$\begin{array}{l} Acc=\frac{TP+TN}{TP+FP+TN+FN} \end{array}$$

**Figure 4:**
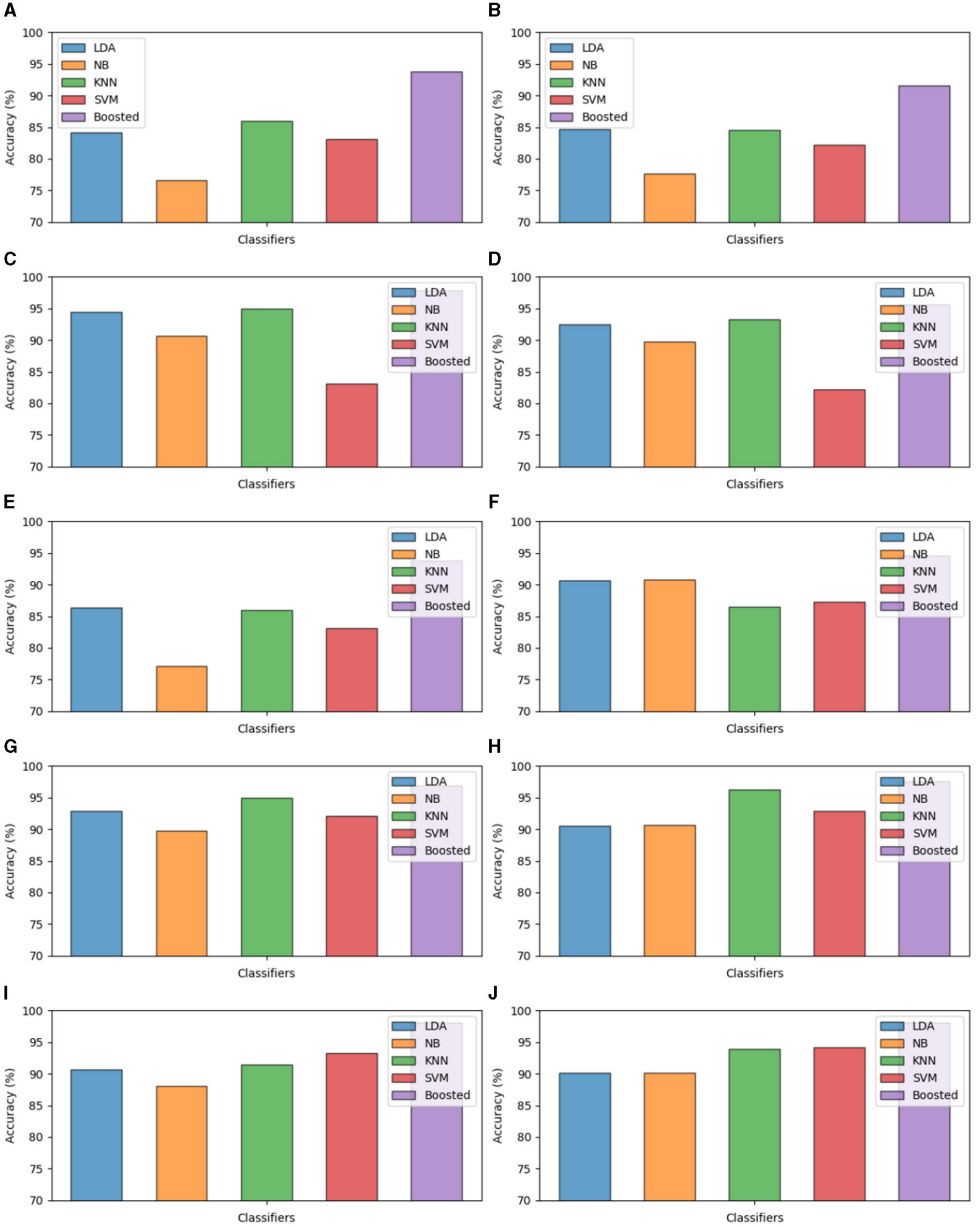
Test accuracy of the classifiers. **(A)** Split ratio balanced data 80:20. **(B)** Split ratio balanced data 70:30. **(C)** Split ratio imbalanced data 80:20. **(D)** Split ratio imbalanced data 70:30. **(E)** CV five-fold balanced data. **(F)** CV 10-fold balanced data. **(G)** CV five-fold imbalanced data. **(H)** CV 10-fold imbalanced data. **(I)** LOSO CV balanced data. **(J)** LOSO imbalanced data.

We conducted an in-depth analysis of various machine learning classifiers into the classification of Dysarthric speech and healthy speech patterns. Utilizing a diverse set of machine learning classifiers, including SVM, Naive Bayes, LDA, KNN, and Boosted Trees, our analysis focused on discerning subtle nuances in speech characteristics. The robust performance of the Boosted Trees classifier, attaining an overall accuracy of 98%, further highlights the potential of machine learning models in effectively distinguishing Dysarthric speech from healthy speech. The split ratios, cross-validation and LOSO-CV test accuracies are presented in Figure 4. These findings contribute valuable insights to the development of accurate diagnostic tools and interventions in the field of speech pathology, promising advancements in personalized healthcare for individuals with speech disorders.

### 4.2 Precison

The precision *(Pre)* metric is a measure of how well a model predicts favorable outcomes. It measures the ratio of true positives (TP) to all predicted positives (FP), which is represented as TP + FP (Equation 8). FP stands for false positives. Calculating this ratio provides the precision value for the model, offering a valuable means to gauge the model's ability to consistently recognize positive instances while concurrently mitigating the occurrence of false positives.


(8) $$\begin{array}{l} Pre=\frac{TP}{TP+FP} \end{array}$$

### 4.3 Recall

The percentage of true positives (TP) over all actual positive instances (TP + FN) is measured by the performance metric recall (*Re*) (Equation 9). It assesses the model's capacity to accurately detect positive cases among all real positive cases. This metric is valuable for evaluating the model's sensitivity in detecting positive cases and is commonly referred to as sensitivity or true positive rate(TPR).


(9) $$\begin{array}{l} Re=\frac{TP}{TP+FN} \end{array}$$

The sensitivit results show that the classifiers are good at being sensitive, giving us useful information about how well each model works. The exceptional sensitivity scores, particularly for SVM and Boosted Trees, hold promising implications for the development of precise diagnostic tools in speech pathology, indicating potential advancements in interventions for individuals with speech disorders.

### 4.4 F1score

The F1 score is a good metric to use, which finds a balance between recall and precision, providing a more complete picture of the model. The F1-score is the weighted average of precision and recall, where the F1 score reaches its best value at 1 (Equation 10).


(10) $$\begin{array}{l} F1=\frac{2*\left(Pre*Re\right)}{Pre+Re} \end{array}$$

The SVM and Boosted Trees classifiers showcased exceptional F1 scores. These outstanding scores underscore the classifiers' adeptness in achieving a harmonious balance between accurately identifying dysarthric speech and minimizing false positives. Furthermore, other classifiers, including Naive Bayes, LDA, and KNN, demonstrated commendable F1 scores respectively. These collective outcomes shed light on the classifiers' effectiveness in achieving a nuanced trade-off between precision and recall, providing valuable insights into the distinctive performance of each model. The exceptional F1 scores, particularly for SVM and Boosted Trees, hold promising implications for the development of refined diagnostic tools in speech pathology, suggesting potential advancements in interventions for individuals with speech disorders.

### 4.5 Area under the receiver operating characteristic curve

AUC, or Area Under the Curve, gives us a general idea of how well a model can tell the difference between the two classes. In our study on Dysarthric speech vs. healthy speech, we evaluated various classifiers, including SVM, Naive Bayes, LDA, KNN, and Boosted Trees. The AUC scores for these classifiers were ranging from 0.89 to 0.991 respectively. These scores indicate the models' overall ability to make a clear distinction between Dysarthric and healthy speech. The higher the AUC score, the better the model is at this discrimination task. This information helps us understand how effective each classifier is in capturing the nuances between the two speech classes, providing a valuable insight into their discriminative capabilities.

### 4.6 Discussion

**Figure 5:**
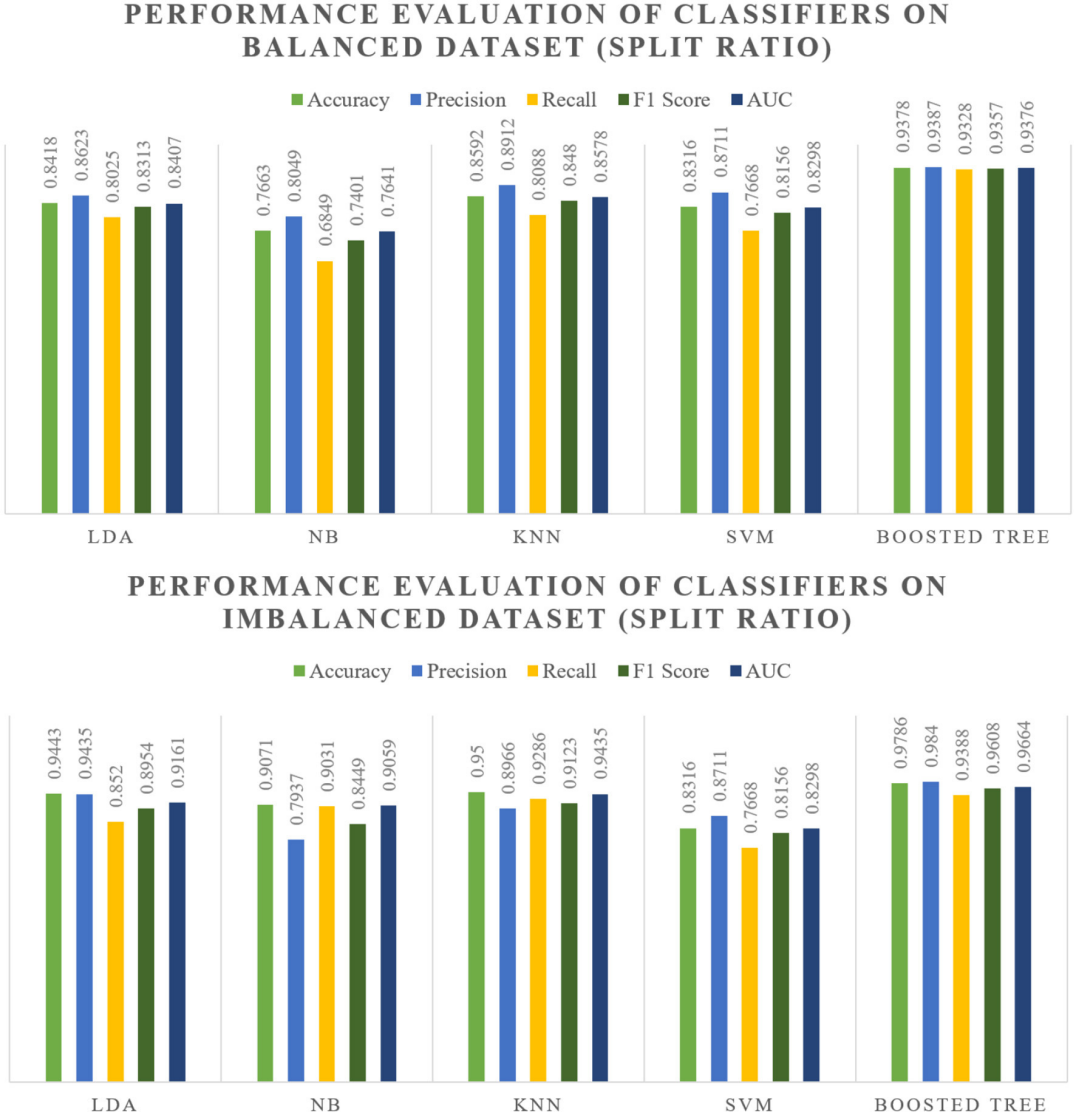
Split ratio performance as shown in Tables 4, 5.

**Figure 6:**
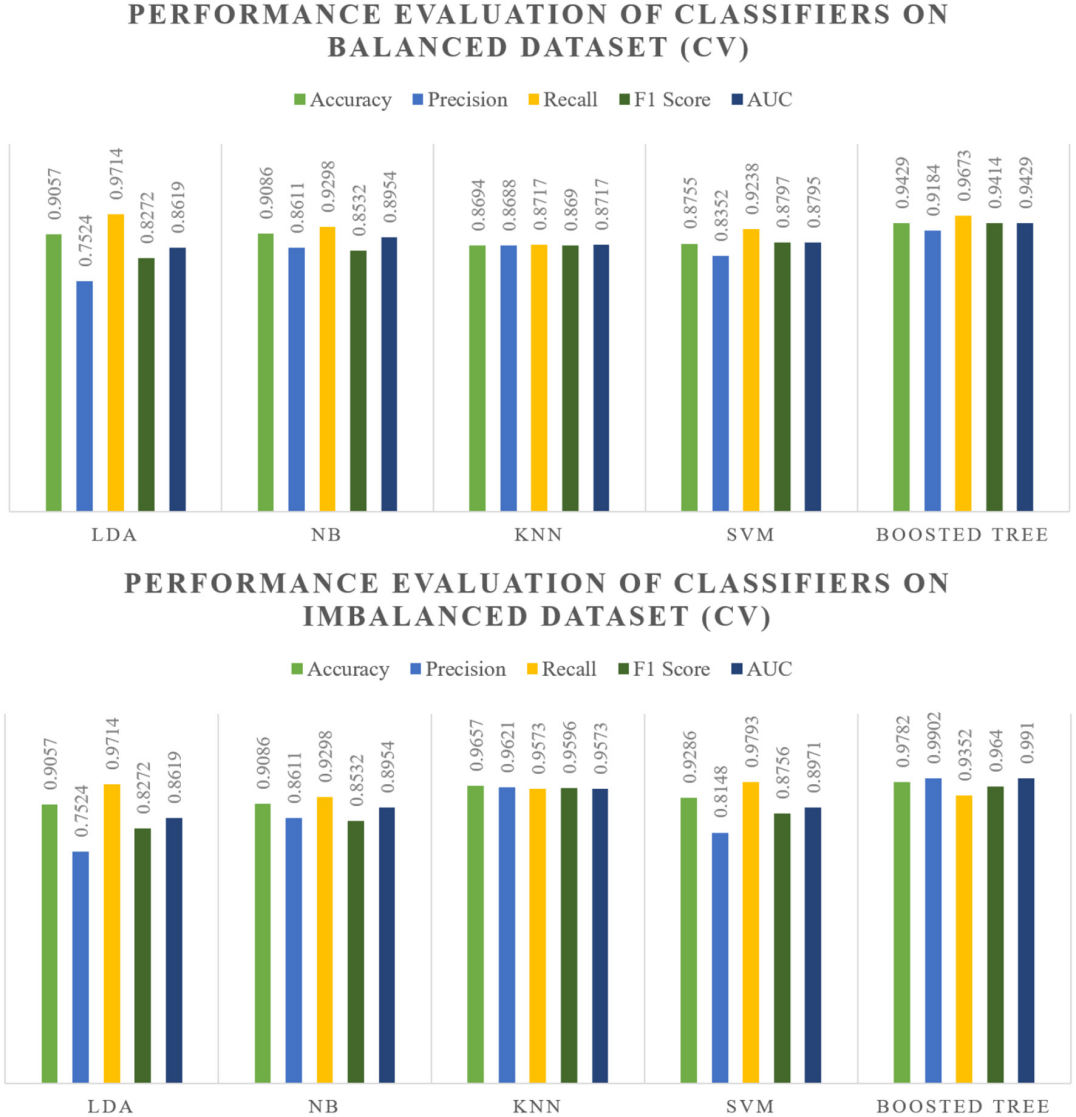
Cross validation performance as shown in Tables 6, 7.

The evaluation of various classifiers on both balanced and imbalanced splits reveals distinct performance trends which are showcased within Figures 5, 6. In the balanced split scenario, the Linear Discriminant Analysis model showcases consistent performance across different data splits (80:20 and 70:30), demonstrating decent precision, recall, and F1 scores around 0.83–0.86, along with notably high Area Under the Curve values.

Naive Bayes exhibits moderate performance, while K-Nearest Neighbors demonstrates relatively good performance, consistently achieving high accuracy, precision, recall, and F1 scores around 0.85–0.89, along with robust AUC values. Support Vector Machines maintain decent performance but with slightly lower AUC values compared to other models. However, the Boosted model consistently outperforms others, displaying significantly high accuracy, precision, recall, and F1 scores around 0.91–0.94 in both balanced and imbalanced splits, along with notably high AUC values, making it a standout performer.

In the context of imbalanced splits, the overall performance trends remain consistent with the balanced splits. Notably, the Boosted model continues to exhibit exceptional performance, showcasing robustness against class imbalances, while SVM's performance shows a relatively smaller difference between balanced and imbalanced scenarios.

Across cross-validation folds (Fold 5 and Fold 10), the models maintain consistent performance trends observed in the split results. Particularly, the Boosted model consistently demonstrates superior performance in both balanced and imbalanced scenarios, showcasing high accuracy, precision, recall, and F1 scores. KNN also maintains commendable performance, establishing itself as a suitable alternative across various scenarios.

This paper integrated advanced methodologies to explore dysarthric and healthy speech classification. Utilizing various machine learning classifiers such as SVM, NB, LDA, KNN, and Boosted Trees, our investigation focused on the multicomponent multitone Amplitude Frequency Modulation (AFM) model designed to encapsulate speech phonemes. We employed the FB-DESA to extract Amplitude Envelope and Instantaneous Frequency components from AFM signals. This unique approach enabled us to analyze the nuanced characteristics of speech phonemes, providing a detailed representation.

For feature extraction, we utilized 28 features from modulated sinusoidal signals, ensuring a comprehensive representation of speech characteristics. After completing extensive feature reduction trials with several feature selection strategies, we assessed the effects of reducing the feature set from 28 to 26 and 24. Despite our attempts, removing features had a significant negative impact on model performance metrics. After removing only two features from the initial set of 28, there is a reduction in accuracy, ranging from 0.2 to 1.5%. Removing four features results in a reduction of accuracy ranging from 1 to 3.5%. Based on these results we conclude all 28 features are essential for the proposed model to produce significant outcomes.

Rigorous testing procedures, including training to testing (80–20 and 70-30) split, cross-validation (five-fold and 10-fold) and LOSO-CV on balanced and imbalanced datasets enhanced the reliability and generalizability of our results. A detailed results of classifier performance across diverse train-test split configurations, aiding in the evaluation of their robustness and efficacy under various dataset distributions are presented in Tables 4, 5. The k-fold cross-validation results presented in Tables 6, 7 to provide a comprehensive evaluation of classifier performance by ensuring that each data point is used for both training and testing, thereby reducing the risk of overfitting and providing a more reliable estimation of model performance. Table 8 showcases the results of Leave-One-Subject-Out Cross-Validation (LOSO-CV) involves iteratively training on all subjects except one and testing on the left-out subject, ensuring each subject serves as a test set exactly once. The reported metrics include precision, recall, and F1-score, providing insights into the classifiers' performance in correctly classifying instances. Our results highlighted notable performances, with the SVM classifier achieving a training accuracy of 96.5% and a test accuracy of 96.7%. Meanwhile, the Boosted Trees classifier showed robust proficiency, boosting an overall accuracy of 97.8%. While k-fold cross-validation consistently yields higher metrics, owing to its aggregated training sets enabling robust model training and generalization, the split ratio shows higher performance metrics without overfitting. Conversely, LOSO-CV exhibits lower performance, attributed to its sensitivity to individual subject variations and potential overfitting, despite its theoretical advantages in utilizing more data. While LOSOCV boasts low bias, as it uses each data point once, its high variance makes it less stable. In contrast to 5-fold and 10-fold cross-validation and different dataset split ratios, LOSO-CV consistently yields lower performance metrics (ranging 2–6%) across various classifiers and dataset configurations. The sensitivity of LOSO-CV to individual subject variations introduces increased variability in training sets, which may lead to challenges in model generalization, particularly in scenarios involving class-specific speech patterns. Furthermore, the computational complexity of LOSO-CV and the potential for overfitting to individual subjects' behavior may further contribute to the observed decrease in classifier performance compared to other methods. These outcomes showed the effectiveness of machine learning models in distinguishing dysarthric from healthy speech, confirming the practicality of our approach.

The integration of advanced feature extraction, sophisticated signal modeling, and powerful machine learning classifiers signifies a significant step forward in dysarthria detection. By exploring how specific signals in speech relate to patterns, our study offers important insights into dysarthria. It also has the potential to improve healthcare tailored to individuals. This work supports field of biomedical engineering, possibly enhancing tools to diagnose speech problems and improving the lives of people with such disorders.

## 5 Comparison results of various approaches

The comparative results of the proposed approach in this work using the modulating features and the existing feature techniques for DYS detection are highlighted in Table 9. This comprehensive comparison involves several significant studies conducted in the categorization field. Al-Qatab and Mustafa (2021) investigated auditory data classification, achieving 95% accuracy through the use of SVM, LDA, and ANN. Vashkevich and Rushkevich (2021) analyzed Vibrato, MFCC, Jitter, and Shimmer features, achieving an 89% accuracy using LDA. Mulfari et al. (2022) concentrated on word recognition using Convolutional Neural Networks (CNN), achieving an impressive 96% accuracy. Meghraoui et al. (2021) employed Support Vector Machines SVM, KNN and RF achieving a 95.6% accuracy in classifying neurological characteristics. Illa et al. (2018) explored supra-segmental traits, achieving 93% accuracy using SVM/DNN. Kodrasi and Bourlard (2019) investigated Weibull distribution, jitter, and shimmer features, achieving 95% accuracy with SVM. Vashkevich et al. (2019) analyzed perturbation analysis, vibrato, jitter, and shimmer traits, achieving 90.7% accuracy using LDA. In contrast, the proposed method incorporated modulating features and employed a diverse set of classifiers such as SVM, Naive Bayes, KNN, LDA, and Boosted Trees, resulting in a significantly higher accuracy of 89%–97.8%. This method demonstrates the importance by integrating a wide array of variables and classifiers, achieving a higher accuracy compared to the individual methodologies explored in other studies.

**Table 9:** Comparison results of various approaches.

**S. No**	**References**	**Features**	**Classifier**	**Accuracy**
1	Al-Qatab and Mustafa (2021)	Acoustic features	SVM, LDA, ANN	95%
	2	Vashkevich and Rushkevich (2021)	Vibrato MFCC Jitter Shimmer	LDA	89%
3		Mulfari et al. (2022)	Isolated word- recognition feature	CNN	96%
	4	Meghraoui et al. (2021)	Neurological features	SVM KNN, RF	95.6%
5		Illa et al. (2018)	Supra-segmental features	SVM/DNN	93%
	6	Kodrasi and Bourlard (2019)	Weibull distribution Jitter Shimmer	SVM	95%
7		Vashkevich et al. (2019)	Perturbation analysis Vibrato Jitter Shimmer	LDA	90.7%
	8	Proposed approach	Modulating features	SVM Naive Bayes KNN LDA Boosted tree	Ranging from 85 to 97.8%

## 6 Conclusion

In conclusion, our thorough investigation into distinguishing dysarthric and healthy speech has shown advanced methods that exhibit promising advancements in speech pathology and personalized healthcare. This work involves the detection of dysarthria by integrating various machine learning classifiers such as SVM, NB, LDA, KNN, and Boosted Trees alongside a novel multicomponent multitone Amplitude Frequency Modulation signal model. The incorporation of the new AFM signal model and the FB-DESA feature extraction method has provided characteristics of speech phonemes, enhancing the complexity of our feature set. A comprehensive analysis, encompassing 28 features derived from modulated sinusoidal signals, along with a robust testing framework employing training to testing (80–20 and 70–30) split, cross-validation (five-fold and 10-fold) and LOSO-CV validates the rigor of our study and the reliability of the results. In this study, various classifiers were evaluated on extracted speech signal features for binary classification. Across varied splits and cross-validation folds, Linear Discriminant Analysis, Naive Bayes, K-Nearest Neighbors, and Support Vector Machines presented commendable accuracies. However, the Boosted model consistently emerged as the top performer, demonstrating superior accuracy across balanced and imbalanced scenarios. This underscores the robustness and efficacy of the Boosted model in discriminating between classes based on speech signal features, suggesting its potential suitability for practical applications. In the future, these models with improved feature sets and network architecture could be used for speech severity assessment, using their promising performance in this domain. Another possible future work may include exploration with increased number of tones and extraction of significant featuresthrough a feature ranking scheme. Additionally, there a scope for further research by evaluating the model on a wider range of datasets.

## Data availability statement

Publicly available datasets were analyzed in this study. This data can be found at: https://www.cs.toronto.edu/~complingweb/data/TORGO/torgo.html; http://www.isle.illinois.edu/sst/data/UASpeech/.

## Author contributions

SS: Data curation, Software, Methodology, Investigation, Writing – original draft. ES: Conceptualization, Validation, Formal analysis, Supervision, Writing – review & editing.
